# Allergen extracts and recombinant proteins: comparison of efficiency of in vitro allergy diagnostics using multiplex assay on a biological microchip

**DOI:** 10.1186/s13223-016-0117-1

**Published:** 2016-03-13

**Authors:** Olga Smoldovskaya, Guzel Feyzkhanova, Alla Arefieva, Sergei Voloshin, Olga Ivashkina, Yuriy Reznikov, Alla Rubina

**Affiliations:** Engelhardt Institute of Molecular Biology, Russian Academy of Sciences (EIMB RAS), 119991 Vavilova str.,32, Moscow, Russia

**Keywords:** Immunoassay, Allergy diagnostics, Microarrays, Recombinant allergens, Biochips

## Abstract

**Background:**

Immunological test systems for diagnostics of type I hypersensitivity involve the following types of antigens: whole allergen extracts, individual highly purified proteins and their recombinant analogues. The goal of this study was to compare the results obtained with whole allergen extracts (birch pollen, cat dander, and timothy grass pollen) and their respective recombinant proteins in biochip-based immunoassay.

**Methods:**

Multiplex fluorescent immunoassay of 139 patients’ blood serum samples was carried out using biological microchips (biochips). sIgE concentrations for the chosen allergens and their recombinant components were measured. ROC analysis was used for comparison of the results and determination of diagnostic accuracy.

**Results:**

The results for the birch pollen extract and its recombinant allergens have shown that the diagnostic accuracy of the methods utilizing the whole allergen extract, its major component Bet v 1 and the combination of major and minor components (Bet v 1 and Bet v 2) was the same. Values for diagnostic accuracy for the cat dander extract and its major recombinant component Fel d 1 were equal. In contrast with birch pollen and cat dander allergens, using of recombinant components of timothy grass pollen (Phl p 1, Phl p 5, Phl p 7 and Phl p 12) did not allow reaching the diagnostic accuracy of using natural extract.

**Conclusions:**

Multiplex analysis of samples obtained from patients with allergy to birch pollen and cat dander using biological microchips has shown that comparable accuracy was observed for the assay with natural extracts and recombinant allergens. In the case of timothy grass allergen, using the recombinant components may be insufficient.

## Background

Immunological test systems for diagnostics of type I hypersensitivity currently involve whole allergen extracts, highly purified allergens from the extracts, and their recombinant analogues obtained by gene engineering techniques. The majority of test systems use extracts and allergen mixtures.

Extracts are heterogeneous protein mixtures isolated from natural materials containing allergenic and non-allergenic material. They allow estimating the reactivity of a patient’s serum toward all potentially allergenic components. Furthermore, allergen extracts contain both species-specific proteins and components with epitopes demonstrating high cross-reactivity with proteins from other origins. This complicates the identification of the primary allergy source [1]. Moreover, the standardization of extracts composition is yet another application problem. It has been shown in a number of studies that allergen extracts produced by number of manufacturers differ considerably in composition and activity [2, 3], leading to discrepancy between analysis results in various test systems [4].

In vitro allergy diagnostics with standardized recombinant allergens and highly purified components of allergen extracts enables getting more reproducible results. It should be noted that the current number of recombinant allergens does not cover the entire spectrum of potentially allergenic proteins present in extracts. This is why the use of only individual protein components is not recommended to identify the allergy source, because it may give a false-negative result in the case of immune response to a protein that is not included in that range. Therefore allergy diagnostics is mostly carried out taking into account clinical history, results of skin tests and/or measurement of sIgE to allergen extracts, whereas molecular-based allergy diagnostics employing individual allergen components is applied for polysensitized patients [5, 6] in order to identify the main sensitizing component, for example, for subsequent allergen specific immunotherapy (ASIT) [7, 8] and for prediction and monitoring of treatment efficiency [9].

Some cases showed that analysis with recombinant allergens increased the diagnostics accuracy [10, 11]. Currently, the most widely used test system based only on recombinant allergens is ImmunoCAP ISAC^®^, manufactured by Phadia (at present trademark of Thermo Fischer Scientific Inc.).

Though individual allergen proteins have been used in studies on the efficacy of diagnostics with recombinant allergens since the 1990s [12], there are a few examples of simultaneous multiplex analysis of sIgE both to whole allergen extracts and their recombinant proteins. It seems that the biological microchip is the most convenient instrument for simultaneous analyzing of sIgE to both individual molecules whole allergen extracts in one serum sample during one assay. The purpose of this work was to compare the diagnostic accuracy of the method for detection of type I hypersensitivity using whole allergen extracts of birch pollen, cat dander and timothy grass pollen (diagnostically significant in central Russia) and their corresponding recombinant proteins on the biological microchips. Comparison of the diagnostic accuracy was made using receiver operating curve (ROC) analysis.

## Methods

### Patients

The study was carried out using patients’ blood serum samples provided by the Federal State Budgetary Institution Polyclinic #1 of the Business Administration for the President of the Russian Federation. Primary selection of the patients was based on the presence of allergy symptoms in the anamnesis: seasonal allergic rhinitis during early spring or summer period as well as year-round rhinitis remissive in outdoor conditions.

Sera from patients demonstrating positive skin prick test (SPT) (reaction >2 mm wheal diameter at least to one of the extracts: birch pollen, timothy grass pollen or cat dander) were chosen for the study. Sera from patients exhibiting positive SPT to the allergen were approved as positive to this allergen. Sera from patients demonstrating negative SPT to the allergen (reaction <2 mm wheal diameter) were approved as negative to this allergen. SPT was performed using salt aqueous extracts, histamine dihydrochloride 0.1 % as a positive control and NaCl 0.9 % as a negative control.

Totally, sera from 139 patients were chosen: 56, 73, and 33 of them were sensitized to birch pollen, cat dander and timothy grass pollen, respectively. The data of the patients’ sera were analyzed anonymously.

### Analysis of blood sera on biochips

**Fig. 1:**
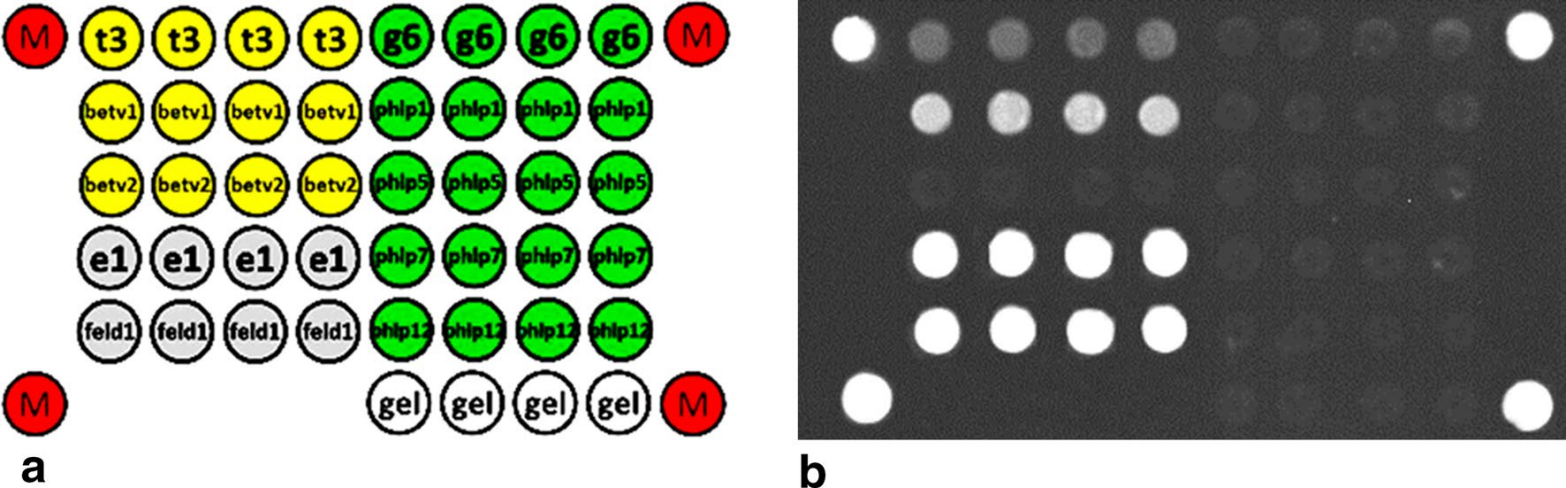
**a** Schematic arrangement of immobilized allergen extracts, their recombinant proteins, and marker spots (М) on biological microchips. Each antigen was immobilized in quadruplicate. Non-specific binding was monitored using spots that contain no proteins (empty gel). **b** An example of fluorescent image of a biochip after immunoassay of a blood serum sample from a patient with allergy to birch pollen and cat dander

Patients’ blood sera were analyzed by the multiplex fluorescent immunoassay method on biological microchips/biochips (EIMB RAS).The biochip manufacturing and procedure of biochip-based immunoassay of allergen-specific IgE have been described previously [13, 14]. Biochip was the set of semispherical hydrogel elements of 0.1 nL containing the following immobilized antigens: birch pollen, recombinant allergens of birch pollen—Bet v 1 and Bet v 2; cat dander, recombinant allergen of cat dander—Fel d 1; timothy grass pollen, recombinant allergens of timothy grass pollen—Phl p 1, Phl p 5, Phl p 7 and Phl p 12 (Fig. 1).

Biochip-based assay allows analyzing of sIgE in the range from 0.15 to 100 IU/ml [12]. We used the concentration 0.35 IU/ml as the cut-off for distinguishing between positive and negative results according to the WHO recommendation.

### Statistical analysis

ROC analysis was performed to estimate the diagnostic accuracy of using allergen extracts and their recombinant proteins. The ROC curves were built and the areas under the curve (AUC) were calculated using MedCalc program, version 15.2.2 [15, 16].When constructing ROC curves for a mixture of recombinant proteins, the result was considered as positive if at least one of the proteins showed positive signal for a chosen cut-off.

## Results and discussion

**Fig. 2:**
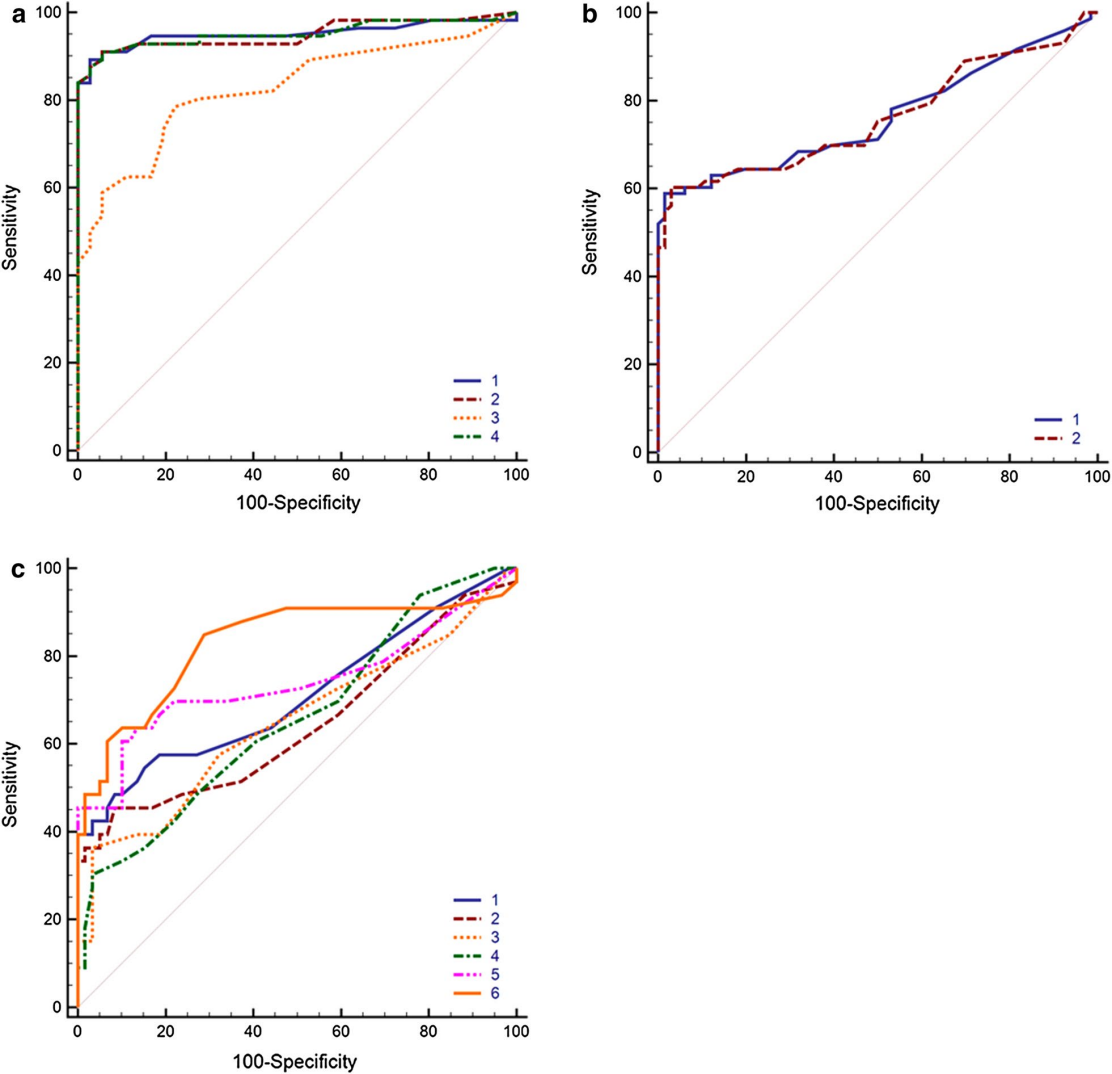
Comparison of ROC curves for methods of identification of type I hypersensitivity using allergen extracts, recombinant components, and combinations of recombinant components. **a** 1-Bet v 1, 2-Bet v 2, 3-Bet v 1 and Bet v 2, 4—birch pollen extract. **b** 1-Fel d 1, 2—cat dander extract and Fel d 1, 3-cat dander extract. **c** 1-Phl p 1, 2-Phl p 5, 3-Phl p 7, 4-Phl p 12, 5-Phl p 1, Phl p 5, Phl p 7, Phl p 12, 6-timothy grass pollen extract

In this study we analyzed 139 blood serum samples from patients with allergy to birch pollen (56 cases), cat dander (73 cases) or timothy grass pollen (33 cases). These allergens are most widespread and diagnostically significant in central Russia. For each allergen we determined the level of sIgE to the natural extracts and to their recombinant proteins: Bet v 1, Bet v 2, Phl p 1, Phl p 5, Phl p 7, Phl p 12, Fel d 1. Fig. 1 shows the biochip structure (a) and the example of fluorescent image after the immunoassay (b) Data were analyzed with ROC analysis. The results are presented in Table 1 and Fig. 2. Statistical analysis of the results for diagnostic accuracy of using the natural birch pollen extract and its recombinant components has shown that the AUC was 0.95 both for the extract and for its major recombinant protein Bet v 1 or 0.83 for the minor component profilin Bet v 2. For the combination Bet v 1 + Bet v 2 the AUC was the same and equaled to 0.95.

**Table 1:** Comparison of ROC analysis results for methods of identification of type I hypersensitivity using allergen extracts, recombinant components, and recombinant components groups

Antigen	AUC	P value
Birch pollen (Positive: 56, Negative: 36)		
Natural extract of birch pollen	0.95	<0.0001
Bet v 1	0.95	<0.0001
Bet v 2	0.83	<0.0001
Bet v 1 + Bet v 2^a^	0.95	<0.0001
Cat dander (Positive: 73, Negative: 66)		
Natural extract of cat dander	0.76	<0.0001
Fel d 1	0.76	<0.0001
Timothy grass pollen (Positive: 33, Negative: 59)		
Natural extract of timothy grass pollen	0.83	<0.0001
Phl p 1	0.71	<0.001
Phl p 5	0.65	<0.02
Phl p 7	0.65	<0.02
Phl p 12	0.66	<0.01
Phl p 1 + Phl p 5	0.71	<0.001
Phl p 1 + Phl p 5 + Phl p 7 + Phl p 12^a^	0.74	<0.0001

^a^For the recombinant proteins groups the sample was considered positive if at least one of the proteins showed positive result for the current cut-off

Analysis of diagnostic accuracy of using the cat dander allergen has shown that the AUC both for the extract and for the recombinant Fel d 1 was the same and equaled to 0.76.

The results obtained for the birch and cat allergens are in accordance with available literature data concerning the frequency of occurrence of immune response to various allergenic components. Sensitization to Bet v 1 as major allergen occurs in a considerable fraction of patients who have allergy to birch pollen (60–90 %) [17], while as little as 12 % of patients have sensitization to other recombinant components (Bet v 2, Bet v 4, Bet v 6, Bet v 8, etc.) [18]. According to various estimates, sensitization to Fel d 1 occurs in 80–95 % of patients with hypersensitivity to cat dander, which results in a high efficiency of the diagnostic test employing this recombinant protein. From the data obtained it can be assumed that for diagnostics purposes allergen extracts can be replaced by a combination of the corresponding recombinant components in case of birch pollen or by the major recombinant protein Fel d 1 for cat dander. However it should be noted that both false positive and false negative results may be obtained in this case. For example, interaction of Fel d 1 allergen with IgE specific to dog dander [19] is observed in 25 % of patients with allergy to dog dander. On the other hand, a patient may show sensitization to minor allergen components that only occur in whole allergen extracts. In these cases, the usage of only recombinant allergens could give false negative results.

To estimate the diagnostic accuracy of analysis in case of allergy to timothy grass, we used natural extract and four different recombinant proteins: Phl p 1, Phl p 5 and Phl p 7, Phl p 12. The obtained data show that the AUC for the extract was 0.83, for Phl p 1 and combination of Phl p 1 and Phl p 5 was 0.71, whereas for Phl p 5, Phl p 7 and Phl p 12 AUC ranged within 0.65–0.66. The AUC was 0.74 for the combination of 4 recombinant components.

Phl p 1 and Phl p 5 are considered to be the major timothy grass pollen components. According to literature data, Phl p 1 is involved in sensitization process for more than 90 % of the patients with timothy grass allergy  [20, 21], so sIgE to Phl p 1 is sufficient for diagnostics of timothy grass pollen allergy for Central European population [22]. However, according to our data, the accuracy of using this recombinant component is lower than using the extract. The addition of the major component Phl p 5, which occurs in up to 80 % of timothy grass pollen sensitized patients, does not increase the AUC value.

The efficiency of the diagnostics with using solely minor cross-reactive components calcium-binding protein Phl p 7 and profilin Phl p 12 is rather poor, but their inclusion in the analysis along with the major proteins improves AUC from 0.71 to 0.74. Thus, it can be concluded that, unlike the situations with birch and cat allergens, the use of available recombinant proteins to timothy grass pollen does not allow reaching the diagnostic accuracy for the natural extract.

In the current work biochips were used as a research instrument for the comparison of diagnostic approaches based on natural extracts and their recombinant components. Furthermore, after modification and validation the biochips could appear as practically significant method combining second- and third-line approaches of allergy diagnostics.

## Conclusions

Multiplex analysis of serum samples obtained from patients with allergy to birch and cat dander carried out on biological microchips has shown that the use of recombinant allergens give accuracy comparable to the natural extracts. In the case of timothy grass, the use of the recombinant components may be insufficient for the identification of allergy source. In some cases, simultaneous analysis using both extracts and their individual protein components is preferable. This approach can be efficiently implemented by means of the protein biochip technology.

## Abbreviations

sIgE: specific immunoglobulin E
ROC: receiver operating curve
AUC: area under curve
ASIT: allergen specific immunotherapy
SPT: skin prick test

## References

[1] Treudler R, Simon JC (2013). Overview of component resolved diagnostics. Curr Allergy Asthma Rep.

[2] Larenas-Linnemann D, Cruz AA, Gutierrez IR, Rodriguez P, Shah-Hosseini K, Michels A (2011). European and Mexican vs US diagnostic extracts of Bermuda grass and cat in skin testing. Ann Allergy Asthma Immunol.

[3] Casset A, Valenta R, Vrtala S (2013). Allergen content and in vivo allergenic activity of house dust mite extracts. Int Arch Allergy Immunol.

[4] Wood RA, Segall N, Ahlstedt S, Williams PB (2007). Accuracy of IgE antibody laboratory results. Ann Allergy Asthma Immunol.

[5] Canonica GW, Ansotegui IJ, Pawankar R, Schmid-Grendelmeier P, van Hage M, Baena-Cagnani CE (2013). A WAO-ARIA-GA²LEN consensus document on molecular-based allergy diagnostics. World Allergy Organ J.

[6] Macchia D, Melioli G, Pravettoni V, Nucera E, Piantanida M, Caminati M (2015). Guidelines for the use and interpretation of diagnostic methods in adult food allergy. Clin Mol Allergy.

[7] Valenta R, Campana R, Marth K, van Hage M (2012). Allergen-specific immunotherapy: from therapeutic vaccines to prophylactic approaches. J Intern Med.

[8] Marth K, Focke-Tejkl M, Lupinek C, Valenta R, Niederberger V (2014). Allergen peptides, recombinant allergens and hypoallergens for allergen-specific immunotherapy. Curr Treat Options Allergy.

[9] Marcucci F, Sensi L, Incorvaia C, Dell’Albani I, Di Cara G, Frati F (2012). Specific IgE response to different grass pollen allergen components in children undergoing sublingual immunotherapy. Clin Mol Allergy.

[10] Maruyama N, Nakagawa T, Ito K, Cabanos C, Borres MP, Movérare R, Tanaka A, Sato S, Ebisawa M (2016). Measurement of specific IgE antibodies to Ses i 1 improves the diagnosis of sesame allergy. Clin Exp Allergy.

[11] Caballero ML, Umpierrez A, Perez-Piñar T, Moneo I, de Burgos C, Asturias JA, Rodríguez-Pérez R (2012). Anisakis simplex recombinant allergens increase diagnosi specificity preserving high sensitivity. Int Arch Allergy Immunol.

[12] Laffer S, Spitzauer S, Susani M, Pairleitner H, Schweiger C, Grönlund H, Menz G, Pauli G, Ishii T, Nolte H, Ebner C, Sehon AH, Kraft D, Eichler HG, Valenta R (1996). Comparison of recombinant timothy grass pollen allergens with natural extract for diagnosis of grass pollen allergy in different populations. J Allergy ClinImmunol.

[13] Rubina AY, Kolchinsky A, Makarov AA, Zasedatelev AS (2008). Why 3-D? Gel-based microarrays in proteomics. Proteomics.

[14] Feyzkhanova GU, Filippova MA, Talibov VO, Dementieva EI, Maslennikov VV, Reznikov YP (2014). Development of hydrogel biochip for in vitro allergy diagnostics. J Immunol Methods.

[15] Fawcett T (2006). An introduction to ROC analysis. Pattern Recogn Lett.

[16] Eusebi P (2013). Diagnostic accuracy measures. Cerebrovasc Dis.

[17] Sekerková A, Poláčková M (2011). Detection of Bet v 1, Bet v 2 and Bet v 4 specific IgE antibodies in the sera of children and adult patients allergic to birch pollen:evaluation of different IgE reactivity profiles depending on age and local sensitization. Int Arch Allergy Immunol.

[18] Rossi RE, Monasterolo G, Monasterolo S (2003). Detection of specific IgE antibodies in the sera of patients allergic to birch pollen using recombinant allergens Bet v 1, Bet v 2, Bet v 4: evaluation of different IgE reactivity profiles. Allergy.

[19] Grönlund H, Saarne T, Gafvelin G, van Hage M (2010). The major cat allergen, Fel d 1, in diagnosis and therapy. Int Arch Allergy Immunol.

[20] Sekerkova A, Polackova M, Striz I (2012). Detection of Phl p 1, Phl p 5, Phl p 7 and Phl p 12 specific IgE antibodies in the sera of children and adult patients allergic to Phleum pollen. Allergol Int..

[21] Rossi RE, Monasterolo G, Monasterolo S (2001). Measurement of IgE antibodies against purified grass-pollen allergens (Phl p 1, 2, 3, 4, 5, 6, 7, 11 and 12) in sera of patients allergic to grass pollen. Allergy.

[22] Bokanovic D, Aberer W, Hemmer W, Heinemann A, Komericki P, Scheffel J, Sturm GJ (2013). Determination of sIgE to rPhl p 1 is sufficient to diagnose grass pollen allergy. Allergy.

